# Efficient simulation of plasma physics’ time fractional modified Korteweg-de Vries equations

**DOI:** 10.1371/journal.pone.0316218

**Published:** 2025-02-12

**Authors:** N. S. Alharthi

**Affiliations:** Department of Mathematics, Faculty of Sciences and Arts, King Abdulaziz University, Rabigh, Saudi Arabia; Kermanshah University of Technology, IRAN, ISLAMIC REPUBLIC OF

## Abstract

In many science and engineering fields, integer-order differential equations are unable to provide a satisfactory explanation for a wide range of phenomena when compared to fractional-order differential equations. The fractional modified Korteweg-de Vries (mKdV) equations are investigated in this work by employing effective analytical methods within the Caputo operator. The findings for the given problems are computed using the Elzaki transformation, the homotopy perturbation method, and the Adomian decomposition method. With these techniques, the problems were first made simpler utilizing the Elzaki transform, and the problems were then comprehensively solved by employing the decomposition and perturbation approaches. A few numerical cases with their approximate analytical solutions are considered to demonstrate the conclusions drawn from the findings. To verify these approaches, we examined two cases and compared them with the real outcomes. By using these methods, the solution to the suggested problem is represented by recurrence relations. The selected issues have series solutions that can be found and have features that more quickly approach the exact results. It is found that there is a strong correlation between the derived results and the real results of every problem when the number of terms increases in the series solution of the problem. The use of efficient techniques that provide higher levels of accuracy with less computation makes the current work innovative. To further benefit the scientific community, the proposed methods can also be used in the future to solve other fractional nonlinear problems.

## 1 Introduction

For a very long time, fractional calculus has been the focus of intensive research. It has been shown that fractional calculus is capable of solving the most challenging real-world issues much more effectively than classical calculus. Newton and Leibniz established the calculus theory in the seventeenth century. The basic concept of fractional calculus arises from the notation of derivatives used by Leibniz in his publications. L’Hopital first raised the question of what the result would be if the derivative order were non-integer [1]. Afterward, the definitions of “derivative” and “integral” were expanded to include any real order. It is interesting to note that simultaneously with these initial theoretical developments, the first practical applications of fractional calculus can also be found. In 1823, Abel analyzed the first application [2, 3], considering the solution of the integral equation related to the tautochrone problem. He found that the solution may be obtained through the use of an integral transform, which is equivalent to a semi-derivative. Thus, Fourier, Liouville, Riemann, Letnikov, Grunwald, etc. provided a great deal of attention to the aforementioned field. The application of fractional-order mathematical modeling has several advantages and important implications. One important component of paradigm adaptation and memory consequences is described by fractional derivatives. Second, details between any two points can be precisely calculated using fractional-order modeling. Fractional orders can be utilized at any point, although the models with integer order simply address the integer event. The models with fractional order describe hereditary and memory aspects of phenomena better than integer-order models [4, 5]. Numerous scholars have presented the definitions of integrations and arbitrary-order derivatives in various manners. The most widely applicable definitions among all of these are those provided by Caputo and Riemann-Liouvilli. The integer derivative reduces to any order by means of a non-integer derivative, so all these methods are just extensions of the methods already in use to handle integer case models [6–8].

Fractional differential equations (FDEs) systematically and effectively represent various natural phenomena [9]. A more accurate description of a real object can be obtained employing this mathematical phenomenon than by using “integer” approaches. Several of these equations perform important roles and serve as tools in mathematics, engineering, physics, dynamical systems, control systems, and other fields in order to enhance the mathematical modeling of multiple physical occurrences. They are also utilized in social science domains such as economics [10], sociology [11] and psychology [12]. Nonlinear partial differential dynamical equations determine the mathematical physics used in physical science. The analytical solutions to these dynamical equations provide the basis for several occurrences in hydrodynamics, plasma physics, fluid mechanics and optics [13–15]. Numerous scholars have been inspired to create novel approaches to solve fractional partial differential equations (FPDEs) with accuracy and precision, and many researchers have created effective direct methods in recent years for the analytical solution of nonlinear FDEs. FPDEs have been solved using a number of efficient techniques, including the Yang transform decomposition method [16], the new extended direct algebraic method [17, 18], the homotopy perturbation Sumudu transform method [19], the fractional variational iteration method [20], the reduced differential transform method [21], the Sine-Gordon expansion method [22], existence and stability analysis [23], the F-expansion method [24], and the Elzaki transform decomposition method [25], along with many others [26–29].

Solitons are currently being examined efficiently. They arise when dispersive effects are eliminated during their propagation. The term “soliton” refers to a solution for the weak massive dispersive nonlinear partial differential equation, which is used in fractional dynamics, engineering, finance, physics, and biology. For soliton solutions, several models are presently under consideration. The Hirota bilinear approach was employed by Liu et al. [30] to derive the solutions for kink and periodic waves. Imran Siddique et al. implemented $\left(\frac{1}{G^{\prime }}\right)$ and modified $\left(\frac{G^{\prime }}{G^{2}}\right)$ to analyze the soliton solutions and other new solutions of fractional complex Ginzburg-Landau equation [31] and time-fractional modified equal-width equation [32]. An improved simple equation technique was used by Arnous et al. [33] to observe the optical soliton solution. The new accurate travelling waves solutions of the nonlinear space-time fractional Sharma-Tasso-Olver equation were extracted by Khush Bukht Mehdi et al. [34] using three integration strategies. Numerous methods are now being considered for the investigation of soliton solutions [35–38].

In order to study waves on shallow water surfaces, Korteweg and de Vries derived the KdV equation in 1895. Numerous studies have been carried out on this perfectly solvable model. Researchers have suggested a number of potential uses for the acoustic waves produced in plasma by crystal lattices and ions. The structure of a typical KdV equation is as follows:

$$\begin{array}{c} B_{t}+6BB_{x}+B_{xxx}=0. \end{array}$$ (1)

The KdV equation has been widely applied in many disciplines, including the analysis of magneto-hydrodynamic waves in heated plasma, shallow water waves, ion acoustic waves, and bubble liquid mixtures [39]. Some theoretical physical features of quantum mechanics are also explained by the KdV model. The strategy is used to generate solitons, shock waves, boundary layer behavior, and turbulence in the domains of fluid dynamics, mass transport, continuum mechanics and aerodynamics. The KdV model has been studied and used for a long time. Numerous modifications and generalizations of the fundamental KdV equation have been made as well [40, 41].

The mKdV equation examined in this study is as follows:

$$\begin{array}{c} D_{t}^{\mu }B(x,t)+6B^{2}\left(x,t\right)B_{x}\left(x,t\right)+B_{xxx}\left(x,t\right)=0,\;\;\;\;\;0<\mu \leq 1. \end{array}$$ (2)

The Elzaki transform decomposition technique (ETDM) and the homotopy perturbation transform method (HPTM) are two unique approaches that are described in this paper. Tarig Elzaki developed the Elzaki transform, which greatly simplifies the time domain solution of ordinary and partial differential equations. Adomian has been working on a numerical method for solving functional equations since the 1980s [42, 43]. It provides the resultant solution as a quickly convergent series that approaches the real results. It is considered a potent technique for handling partial and differential equations of both integer and non-integer order that are linear and nonlinear, homogeneous and nonhomogeneous. He first proposed the homotopy perturbation method (HPM) in 1998 [44], and it was later improved and refined [45, 46]. It also provides the resultant solution as a quickly convergent series that approaches the real results. In summary, the initial FPDE is converted into its corresponding partial differential equation by approximating the fractional Caputo derivative using the Elzaki transform approach. The initial FPDE can be quickly and easily solved using the homotopy perturbation approach and the Adomian decomposition method for the resulting partial differentiation equation. As a useful and efficient mathematical tool for solving nonlinear equations, it usually only requires one iteration to produce a highly precise solution.

This study is presented in the following manner: definitions of fractional derivatives and the background of the Elzaki transform technique are provided in section 2. Sections 3 and 4 discuss the FDE application model using the chosen methods. A convergence study of the recommended strategies is offered in section 5. FDEs are solved in section 6. Section 7 discusses the results, including numerical simulations and graphical comparisons, to validate the accuracy of the proposed methods. Finally, Section 8 contains our concluding remarks.

## 2 Preliminaries

This section offers some fundamental definitions related to our present work.

### 2.1 Defintion

The Riemann-Liouville’s (RL) derivative operator is given by [47–49]

$$\begin{array}{c} D^{\mu }B\left(ℵ\right)=\{\begin{array}{c} \frac{d^{\varrho }}{d{ℵ}^{\varrho }}B\left(ℵ\right),\;\;\mu =\varrho ,\\ \frac{1}{\Gamma (\varrho -\mu )}\frac{d}{d{ℵ}^{\varrho }}\int_{0}^{ℵ}\frac{B(ℵ)}{{\left(ℵ-\delta \right)}^{\mu -\varrho +1}}d\delta ,\;\;\varrho -1<\mu <\varrho , \end{array} \end{array}$$

with $\varrho \in Z^{+}$, *μ* ∈ *R*^+^ and

$$\begin{array}{c} D^{-\mu }B\left(ℵ\right)=\frac{1}{\Gamma (\mu )}\int_{0}^{ℵ}{\left(ℵ-\delta \right)}^{\mu -1}B\left(\delta \right)d\delta ,\;\;0<\mu \leq 1. \end{array}$$

### 2.2 Defintion

The RL fractional integral operator is given by [47–49]

$$\begin{array}{c} J^{\mu }B\left(ℵ\right)=\frac{1}{\Gamma (\mu )}\int_{0}^{ℵ}{\left(ℵ-\delta \right)}^{\mu -1}B\left(ℵ\right)dℵ,\;\;ℵ>0,\;\;\mu >0, \end{array}$$

and has the following properties:

$$\begin{array}{c} \begin{array}{c} J^{\mu }{ℵ}^{\varrho }=\frac{\Gamma (\varrho +1)}{\Gamma (\varrho +\mu +1)}{ℵ}^{\varrho +\delta },\\ D^{\mu }{ℵ}^{\varrho }=\frac{\Gamma (\varrho +1)}{\Gamma (\varrho -\mu +1)}{ℵ}^{\varrho -\delta }. \end{array} \end{array}$$

### 2.3 Defintion

The Caputo fractional derivative is given by [47–49]

$$\begin{array}{c} D^{\mu }B\left(ℵ\right)=\{\begin{array}{c} \frac{1}{\Gamma (\varrho -\mu )}\int_{0}^{ℵ}\frac{B^{\varrho }\left(\delta \right)}{{\left(ℵ-\delta \right)}^{\mu -\varrho +1}}d\delta ,\;\;\varrho -1<\mu <\varrho ,\\ \frac{d^{\varrho }}{d{ℵ}^{\varrho }}B\left(ℵ\right),\;\;\varrho =\mu , \end{array} \end{array}$$ (3)

and has the following properties:

$$\begin{array}{c} \begin{array}{cc} J_{ℵ}^{\mu }D_{ℵ}^{\mu }B\left(ℵ\right) & =g\left(ℵ\right)-\sum_{k=0}^{m}g^{k}\left(0^{+}\right)\frac{{ℵ}^{k}}{k!},\;\;for\;\;ℵ>0,\;\;and\;\;\varrho -1<\mu \leq \varrho ,\;\;\varrho \in N,\\ D_{ℵ}^{\mu }J_{ℵ}^{\mu }B\left(ℵ\right) & =g(ℵ). \end{array} \end{array}$$ (4)

### 2.4 Defintion

The Elzaki transform (ET) of the function B(Π) is given by [50]

$$\begin{array}{c} E\left\{B\left(\Pi \right)\right\}=M\left(u\right)=u\int_{u}^{\infty }e^{\frac{-\Pi }{u}}B\left(\Pi \right)d\Pi ,\;\;\Pi >0. \end{array}$$ (5)

### 2.5 Defintion

The ET of Caputo derivative is given by [51]

$$\begin{array}{c} E\left[D_{ℵ}^{\mu }B\left(ℵ\right)\right]=u^{-\mu }E\left[B\left(ℵ\right)\right]-\sum_{i=0}^{n-1}u^{2-\mu +i}B^{\left(i\right)}\left(0\right),\;\;where\;\;n-1<\mu <n. \end{array}$$

Some properties of the ET are as follows:

1. *E*[Π^*n*^] = *n*!*u*^*n*+2^.
2.
$E\left[B^{\prime }\right]=\frac{M(u)}{u}-uB\left(0\right).$

3.
$E\left[B^{\prime \prime }\right]=\frac{M(u)}{u^{2}}-B\left(0\right)-uB^{\prime }\left(0\right).$

4.
$E\left[B^{n}\right]=\frac{M(u)}{u^{2}}-\sum_{i=0}^{n-1}u^{2-n+i}B^{\left(i\right)}\left(0\right).$

## 3 Road map of the HPTM

This section offers the general implementation of the HPTM to handle the FPDE are as given:

$$\begin{array}{c} D_{\Pi }^{\mu }B\left(ℵ,\Pi \right)=\left[D_{1}+E_{1}\right]B\left(ℵ,\Pi \right),\;\;ℵ,\Pi >0\;\;0<\mu \leq 1, \end{array}$$ (6)

with

$$\begin{array}{c} B(ℵ,0)=\vartheta (ℵ). \end{array}$$

Here, $D_{\Pi }^{\mu }=\frac{\partial^{\mu }}{\partial \Pi^{\mu }}$ indicating the Caputo derivative having order *μ*, and $D_{1}\left(ℵ,\Pi \right),$ $D_{1}\left(ℵ,\Pi \right)$ are linear and nonlinear operators.

Operating Definition 2.5 at n = 1, we may have the following:

$$\begin{array}{c} \frac{1}{u^{\mu }}\left\{M\left(u\right)-u^{2}B\left(ℵ,0\right)\right\}=E\left[\left[D_{1}+E_{1}\right]B\left(ℵ,\Pi \right)\right]. \end{array}$$ (7)

Thus, we obtain the following:

$$\begin{array}{c} M\left(u\right)=u^{2}B\left(ℵ,0\right)+u^{\mu }E\left[\left[D_{1}+E_{1}\right]B\left(ℵ,\Pi \right)\right]. \end{array}$$ (8)

Here M(u)=E[B(ℵ,Π)].

On operating inverse ET, we may have the following:

$$\begin{array}{c} B\left(ℵ,\Pi \right)=B\left(ℵ,0\right)+E^{-1}\left[u^{\mu }E\left[\left[D_{1}+E_{1}\right]B\left(ℵ,\Pi \right)\right]\right]. \end{array}$$ (9)

By utilizing HPM, we may obtain the following:

$$\begin{array}{c} B\left(ℵ,\Pi \right)=\sum_{m=0}^{\infty }\epsilon^{m}B_{m}\left(ℵ,\Pi \right). \end{array}$$ (10)

Here, *ϵ* ∈ [0, 1] is homotopy parameter.

The nonlinear term is illustrated as

$$\begin{array}{c} \;G_{1}\left[B\left(ℵ,\Pi \right)\right]=\sum_{n=0}^{\infty }\epsilon^{n}H_{n}\left(ℵ,\Pi \right), \end{array}$$ (11)

and is determined as follows:

$$\begin{array}{c} \;H_{n}\left(ℵ,\Pi \right)=\frac{1}{\Gamma (n+1)}D_{\epsilon }^{k}{\left[E_{1}(\sum_{n=0}^{\infty }\epsilon^{n}B_{n})\right]}_{\epsilon =0}, \end{array}$$ (12)

with $D_{\epsilon }^{n}=\frac{\partial^{n}}{\partial \epsilon^{n}}.$

Utilizing (10) and (11) in (9), we may get the following:

$$\begin{array}{c} \;\sum_{n=0}^{\infty }\epsilon^{n}B_{n}\left(ℵ,\Pi \right)=B\left(ℵ,0\right)+\epsilon \times (E^{-1}[u^{\mu }E\left\{D_{1}\sum_{n=0}^{\infty }\epsilon^{n}B_{n}\left(ℵ,\Pi \right)+\sum_{n=0}^{\infty }\epsilon^{n}H_{n}\left(ℵ,\Pi \right)\right\}]). \end{array}$$ (13)

Equating the *ϵ* coefficient on both sides as demonstrated below:

$$\begin{array}{c} \;\begin{array}{cc} & \epsilon^{0}:B_{0}\left(ℵ,\Pi \right)=B\left(ℵ,0\right),\\ & \epsilon^{1}:B_{1}\left(ℵ,\Pi \right)=E^{-1}[u^{\mu }E\left(F_{1}\left(B_{0}\left(ℵ,\Pi \right)\right)+H_{0}\left(ℵ,\Pi \right)\right)],\\ & \epsilon^{2}:B_{2}\left(ℵ,\Pi \right)=E^{-1}[u^{\mu }E\left(F_{1}\left(B_{1}\left(ℵ,\Pi \right)\right)+H_{1}\left(ℵ,\Pi \right)\right)],\\ & .\\ & .\\ & .\\ & \epsilon^{n}:B_{n}\left(ℵ,\Pi \right)=E^{-1}[u^{\mu }E\left(F_{1}\left(B_{n-1}\left(ℵ,\Pi \right)\right)+H_{n-1}\left(ℵ,\Pi \right)\right)],\;\;n>0,n\in N. \end{array} \end{array}$$ (14)

Hence, our approximate solution may be written in series form as:

$$\begin{array}{c} \;B\left(ℵ,\Pi \right)=\underset{M\to \infty }{\text{lim}}\sum_{n=1}^{M}B_{n}\left(ℵ,\Pi \right). \end{array}$$ (15)

## 4 Road map of the ETDM

This section offers the general implementation of the ETDM to handle the FPDE as outlined:

$$\begin{array}{c} D_{\Pi }^{\mu }B(ℵ,\Pi )=\left[D_{1}+E_{1}\right]B\left(ℵ,\Pi \right),\;\;ℵ,\Pi >0\;\;0<\mu \leq 1, \end{array}$$ (16)

with

$$\begin{array}{c} B(ℵ,0)=\vartheta (ℵ). \end{array}$$

Here, $D_{\Pi }^{\mu }=\frac{\partial^{\mu }}{\partial \Pi^{\mu }}$ indicates the Caputo derivative with order *μ*, and $D_{1}$, $E_{1}$ are linear and non-linear operators.

Operating Definition 2.5 at n = 1, we may have the following:

$$\begin{array}{c} \begin{array}{cc} & \frac{1}{u^{\mu }}\left\{M\left(u\right)-u^{2}B\left(ℵ,0\right)\right\}=E\left[\left[D_{1}+E_{1}\right]B\left(ℵ,\Pi \right)\right]. \end{array} \end{array}$$ (17)

Thus, we obtain the following:

$$\begin{array}{c} M\left(u\right)=uB\left(ℵ,0\right)+u^{\mu }E\left[\left[D_{1}+E_{1}\right]B\left(ℵ,\Pi \right)\right]. \end{array}$$ (18)

Here, M(u)=E[B(ℵ,Π)].

On operating inverse ET, we may have the following:

$$\begin{array}{c} B\left(ℵ,\Pi \right)=B\left(ℵ,0\right)+E^{-1}[u^{\mu }E\left[\left[D_{1}+E_{1}\right]B\left(ℵ,\Pi \right)\right]. \end{array}$$ (19)

Thus, the solution may be written in an infinite series form as follows:

$$\begin{array}{c} B\left(ℵ,\Pi \right)=\sum_{n=0}^{\infty }B_{n}\left(ℵ,\Pi \right). \end{array}$$ (20)

The nonlinear term is illustrated:

$$\begin{array}{c} E_{1}\left[B\left(ℵ,\Pi \right)\right]=\sum_{n=0}^{\infty }A_{n}\left(ℵ,\Pi \right), \end{array}$$ (21)

with

$$\begin{array}{c} A_{n}\left(ℵ,\Pi \right)=\frac{1}{n!}{\left[\frac{\partial^{n}}{\partial \ell^{n}}\{E_{1}(\sum_{n=0}^{\infty }\ell^{n}B_{n})\}\right]}_{\ell =0},\;\;n=0,1,2,\cdots \end{array}$$ (22)

Utilizing (19) and (20) in (18), we may get

$$\begin{array}{c} \sum_{n=0}^{\infty }B_{n}\left(ℵ,\Pi \right)=B\left(ℵ,0\right)+E^{-1}u^{\mu }[E\{D_{1}(\sum_{n=0}^{\infty }B_{n}\left(ℵ,\Pi \right))+\sum_{n=0}^{\infty }A_{n}\left(ℵ,\Pi \right)\}]. \end{array}$$ (23)

On equating both sides we obtain the following:

$$\begin{array}{c} B_{0}\left(ℵ,\Pi \right)=B\left(ℵ,0\right), \end{array}$$ (24)

$$\begin{array}{c} B_{1}\left(ℵ,\Pi \right)=E^{-1}[u^{\mu }E\left\{F_{1}\left(B_{0}\left(ℵ,\Pi \right)\right)+A_{0}\left(ℵ,\Pi \right)\right\}]. \end{array}$$

Hence, the general solution for *m* ≥ 1 is demonstrated as follows:

$$\begin{array}{c} B_{m+1}\left(ℵ,\Pi \right)=E^{-1}[u^{\mu }E\left\{F_{1}\left(B_{m}\left(ℵ,\Pi \right)\right)+A_{m}\left(ℵ,\Pi \right)\right\}]. \end{array}$$

## 5 Convergence analysis

This section offers the convergence analysis of the suggested techniques.

### 5.1 Theorem

Assume that ℑ(ℵ, Π) is the accurate solution of (6) and let ℑ(ℵ, Π), ℑ_*n*_(ℵ, Π) ∈ *H* and *ε* ∈ (0, 1), here H indicates the Hilbert space. The result gained $\sum_{q=0}^{\infty }{ℑ}_{q}\left(ℵ,\Pi \right)$ approach ℑ(ℵ, Π) if ℑ_*q*_(ℵ, Π) ≤ ℑ_*q*−1_(ℵ, Π) ∀*q* > *A*, i.e., for all *ω* > 0∃*A* > 0, such that ||ℑ_*q* + *n*_(ℵ, Π)|| ≤ *θ*, ∀*m*, *n* ∈ *N*. Proof. We assume a sequence $\sum_{q=0}^{\infty }{ℑ}_{q}\left(ℵ,\Pi \right).$

$$\begin{array}{c} \begin{array}{cc} C_{0}\left(ℵ,\Pi \right)= & {ℑ}_{0}\left(ℵ,\Pi \right),\\ C_{1}\left(ℵ,\Pi \right)= & {ℑ}_{0}\left(ℵ,\Pi \right)+{ℑ}_{1}\left(ℵ,\Pi \right),\\ C_{2}\left(ℵ,\Pi \right)= & {ℑ}_{0}\left(ℵ,\Pi \right)+{ℑ}_{1}\left(ℵ,\Pi \right)+{ℑ}_{2}\left(ℵ,\Pi \right),\\ C_{3}\left(ℵ,\Pi \right)= & {ℑ}_{0}\left(ℵ,\Pi \right)+{ℑ}_{1}\left(ℵ,\Pi \right)+{ℑ}_{2}\left(ℵ,\Pi \right)+{ℑ}_{3}\left(ℵ,\Pi \right),\\ & \vdots \\ C_{q}\left(ℵ,\Pi \right)= & {ℑ}_{0}\left(ℵ,\Pi \right)+{ℑ}_{1}\left(ℵ,\Pi \right)+{ℑ}_{2}\left(ℵ,\Pi \right)+\cdots +{ℑ}_{q}\left(ℵ,\Pi \right). \end{array} \end{array}$$ (25)

We should prove that *C*_*q*_(ℵ, Π) forms a “Cauchy sequence”. Also, assume the following:

$$\begin{array}{c} \begin{array}{cc} \left|\right|C_{q+1}\left(ℵ,\Pi \right)-C_{q}\left(ℵ,\Pi \right)\left|\right| & =\left|\right|{ℑ}_{q+1}\left(ℵ,\Pi \right)||\leq \varepsilon ||{ℑ}_{q}\left(ℵ,\Pi \right)||\leq \varepsilon^{2}\left|\right|{ℑ}_{q-1}\left(ℵ,\Pi \right)||\leq \varepsilon^{3}\left|\right|{ℑ}_{q-2}\left(ℵ,\Pi \right)\left|\right|\cdots \\ & \leq \varepsilon_{q+1}\left|\right|{ℑ}_{0}\left(ℵ,\Pi \right)\left|\right|. \end{array} \end{array}$$ (26)

For *q*, *n* ∈ *N*, we may have the following:

$$\begin{array}{c} \begin{array}{cc} \left|\right|C_{q}\left(ℵ,\Pi \right)-C_{n}\left(ℵ,\Pi \right)\left|\right|= & \left|\right|{ℑ}_{q+n}\left(ℵ,\Pi \right)||=\left|\right|C_{q}\left(ℵ,\Pi \right)-C_{q-1}\left(ℵ,\Pi \right)+\left(C_{q-1}\left(ℵ,\Pi \right)-C_{q-2}\left(ℵ,\Pi \right)\right)\\ & +\left(C_{q-2}\left(ℵ,\Pi \right)-C_{q-3}\left(ℵ,\Pi \right)\right)+\cdots +\left(C_{n+1}\left(ℵ,\Pi \right)-C_{n}\left(ℵ,\Pi \right)\right)\left|\right|\\ \leq & \left|\right|C_{q}\left(ℵ,\Pi \right)-C_{q-1}\left(ℵ,\Pi \right)||+\left|\right|\left(C_{q-1}\left(ℵ,\Pi \right)-C_{q-2}\left(ℵ,\Pi \right)\right)\left|\right|\\ & +\left|\right|\left(C_{q-2}\left(ℵ,\Pi \right)-C_{q-3}\left(ℵ,\Pi \right)\right)\left|\right|+\cdots +\left|\right|\left(C_{n+1}\left(ℵ,\Pi \right)-C_{n}\left(ℵ,\Pi \right)\right)\left|\right|\\ \leq & \varepsilon^{q}\left|\right|{ℑ}_{0}\left(ℵ,\Pi \right)||+\varepsilon^{q-1}\left|\right|{ℑ}_{0}\left(ℵ,\Pi \right)||+\cdots +\varepsilon^{q+1}\left|\right|{ℑ}_{0}\left(ℵ,\Pi \right)\left|\right|\\ = & \left|\right|{ℑ}_{0}\left(ℵ,\Pi \right)\left|\right|\left(\varepsilon^{q}+\varepsilon^{q-1}+\varepsilon^{q+1}\right)\\ = & \left|\right|{ℑ}_{0}\left(ℵ,\Pi \right)\left|\right|\frac{1-\varepsilon^{q-n}}{1-\varepsilon^{q+1}}\varepsilon^{n+1}. \end{array} \end{array}$$ (27)

As 0 < *ε* < 1, and ℑ_0_(ℵ, Π) are bound, so take *θ* = 1 − *ε*/(1 − *ε*_*q*−*n*_)*ε*^*n*+1^||ℑ_0_(ℵ, Π)||, and we get

$$\begin{array}{c} \left|\right|{ℑ}_{q+n}\left(ℵ,\Pi \right)\left|\right|\leq \theta ,\forall q,n\in N. \end{array}$$ (28)

Thus, ${\left\{{ℑ}_{q}\left(ℵ,\Pi \right)\right\}}_{q=0}^{\infty }$ forms “Cauchy sequence” in H. It showed that ${\left\{{ℑ}_{q}\left(ℵ,\Pi \right)\right\}}_{q=0}^{\infty }$ is a convergent sequence with the limit lim_*q* → ∞_ℑ_*q*_(ℵ, Π) = ℑ(ℵ, Π) for ∃ℑ(ℵ,Π)∈H which is proved.

### 5.2 Theorem

Assume $\sum_{h=0}^{k}{ℑ}_{h}\left(ℵ,\Pi \right)$ is finite and ℑ(ℵ, Π) indicates the series form solution. Suppose *ε* > 0 with ||ℑ_*h*+1_(ℵ, Π)|| ≤ ||ℑ_*h*_(ℵ, Π)||, the maximum absolute error is given below.

$$\begin{array}{c} ||ℑ\left(ℵ,\Pi \right)-\sum_{h=0}^{k}{ℑ}_{h}\left(ℵ,\Pi \right)||<\frac{\varepsilon^{k+1}}{1-\varepsilon }\left|\right|{ℑ}_{0}\left(ℵ,\Pi \right)\left|\right|. \end{array}$$ (29)

Proof. Assume $\sum_{h=0}^{k}{ℑ}_{h}\left(ℵ,\Pi \right)$ is finite which shows that $\sum_{h=0}^{k}{ℑ}_{h}\left(ℵ,\Pi \right)<\infty$.

Consider the following:

$$\begin{array}{c} \begin{array}{cc} ||ℑ\left(ℵ,\Pi \right)-\sum_{h=0}^{k}{ℑ}_{h}\left(ℵ,\Pi \right)\left|\right|= & \left|\right|\sum_{h=k+1}^{\infty }{ℑ}_{h}\left(ℵ,\Pi \right)\left|\right|\\ \leq & \sum_{h=k+1}^{\infty }\left|\right|{ℑ}_{h}\left(ℵ,\Pi \right)\left|\right|\\ \leq & \sum_{h=k+1}^{\infty }\varepsilon^{h}\left|\right|{ℑ}_{0}\left(ℵ,\Pi \right)\left|\right|\\ \leq & \varepsilon^{k+1}\left(1+\varepsilon +\varepsilon^{2}+\cdots \right)\left|\right|{ℑ}_{0}\left(ℵ,\Pi \right)\left|\right|\\ \leq & \frac{\varepsilon^{k+1}}{1-\varepsilon }\left|\right|{ℑ}_{0}\left(ℵ,\Pi \right)\left|\right|. \end{array} \end{array}$$ (30)

which is proved.

### 5.3 Theorem

The solution of (16) is unique at $0<\left(\varsigma_{1}+\varsigma_{2}\right)\left(\frac{\Pi^{\mu }}{\Gamma (\mu +1)}\right)<1.$

Proof: Let *H* = (*C*[*J*], ||.||) with the norm ||*ϕ*(Π)|| = *max*_Π∈*J*_|*ϕ*(Π)| is Banach space, ∀ continuous function on *J*. Let *I*: *H* → *H* is a non-linear mapping, where

$$\begin{array}{c} B_{l+1}^{C}=B_{0}^{C}+E^{-1}\left[u^{\mu }E\left[D_{1}\left(B_{l}\left(ℵ,\Pi \right)\right)+E_{1}\left(B_{l}\left(ℵ,\Pi \right)\right)\right]\right],\;\;l\geq 0. \end{array}$$

Suppose that $|D_{1}\left(B\right)-D_{1}\left(B^{*}\right)|<\varsigma_{1}\left|B-B^{*}\right|$ and $|E_{1}\left(B\right)-E_{1}\left(B^{*}\right)|<\varsigma_{2}\left|B-B^{*}\right|$, where B:=B(ℵ,Π) and $B^{*}:=B^{*}\left(ℵ,\Pi \right)$ are two different function values and $\varsigma_{1}$,$\varsigma_{2}$ are Lipschitz constants.

$$\begin{array}{c} \begin{array}{cc} ||IB-IB^{*}\left|\right| & \leq max_{t\in J}|E^{-1}[u^{\mu }E\left[D_{1}\left(B\right)-D_{1}\left(B^{*}\right)\right]\\ & +u^{\mu }E\left[E_{1}\left(B\right)-E_{1}\left(B^{*}\right)\right]\left|\right]\\ & \leq max_{\Pi \in J}[\varsigma_{1}E^{-1}[u^{\mu }E[|B-B^{*}\left|]\right]\\ & +\varsigma_{2}E^{-1}[u^{\mu }E[|B-B^{*}\left|]]\right]\\ & \leq max_{t\in J}\left(\varsigma_{1}+\varsigma_{2}\right)[E^{-1}[u^{\mu }E|B-B^{*}\left|\right]]\\ & \leq \left(\varsigma_{1}+\varsigma_{2}\right)[E^{-1}[u^{\mu }E||B-B^{*}\left||\right]]\\ & =\left(\varsigma_{1}+\varsigma_{2}\right)\left(\frac{\Pi^{\mu }}{\Gamma (\mu +1)}\right)||B-B^{*}\left|\right| \end{array} \end{array}$$ (31)

I is contraction as $0<\left(\varsigma_{1}+\varsigma_{2}\right)\left(\frac{\Pi^{\mu }}{\Gamma (\mu +1)}\right)<1$. From the above theorem the solution of (16) is unique.

### 5.4 Theorem

The solution of (16) is convergent.

Proof: Let $B_{m}=\sum_{r=0}^{m}B_{r}\left(ℵ,\Pi \right)$. To prove that $B_{m}$ is a Cauchy sequence in H. Assume,

$$\begin{array}{c} \begin{array}{cc} \left|\right|B_{m}-B_{n}\left|\right| & =max_{\Pi \in J}\left|\sum_{r=n+1}^{m}B_{r}\right|,\;\;n=1,2,3,\cdots \\ & \leq max_{\Pi \in J}|E^{-1}[u^{\mu }E[\sum_{r=n+1}^{m}\left(D_{1}\left(B_{r-1}\right)+E_{1}\left(B_{r-1}\right)\right)]]|\\ & =max_{\Pi \in J}|E^{-1}[u^{\mu }E[\sum_{r=n+1}^{m-1}\left(D_{1}\left(B_{r}\right)+E_{1}\left(B_{r}\right)\right)]]|\\ & \leq max_{\Pi \in J}\left|E^{-1}\left[u^{\mu }E\left[\left(D_{1}\left(B_{m-1}\right)-D_{1}\left(B_{n-1}\right)+E_{1}\left(B_{m-1}\right)-E_{1}\left(B_{n-1}\right)\right)\right]\right]\right|\\ & \leq \varsigma_{1}max_{\Pi \in J}\left|E^{-1}\left[u^{\mu }E\left[\left(D_{1}\left(B_{m-1}\right)-D_{1}\left(B_{n-1}\right)\right)\right]\right]\right|\\ & +\varsigma_{2}max_{\Pi \in J}\left|E^{-1}\left[u^{\mu }E\left[\left(E_{1}\left(B_{m-1}\right)-E_{1}\left(B_{n-1}\right)\right)\right]\right]\right|\\ & =\left(\varsigma_{1}+\varsigma_{2}\right)\left(\frac{\Pi^{\mu }}{\Gamma (\mu +1)}\right)\left|\right|B_{m-1}-B_{n-1}\left|\right| \end{array} \end{array}$$ (32)

Let *m* = *n* + 1, then

$$\begin{array}{c} \begin{array}{c} \left|\right|B_{n+1}-B_{n}\left|\right|\leq \varsigma ||B_{n}-B_{n-1}\left|\right|\leq \varsigma^{2}\left|\right|B_{n-1}B_{n-2}\left|\right|\leq \cdots \leq \varsigma^{n}\left|\right|B_{1}-B_{0}\left|\right|, \end{array} \end{array}$$ (33)

where $\varsigma =\left(\varsigma_{1}+\varsigma_{2}\right)\left(\frac{\Pi^{\mu }}{\Gamma (\mu +1)}\right)$. Similarly, we have

$$\begin{array}{c} \begin{array}{cc} \left|\right|B_{m}-B_{n}\left|\right| & \leq \left|\right|B_{n+1}-B_{n}\left|\right|+\left|\right|B_{n+2}B_{n+1}\left|\right|+\cdots +\left|\right|B_{m}-B_{m-1}\left|\right|,\\ & \left(\varsigma^{n}+\varsigma^{n+1}+\cdots +\varsigma^{m-1}\right)\left|\right|B_{1}-B_{0}\left|\right|\\ & \leq \varsigma^{n}(\frac{1-\varsigma^{m-n}}{1-\varsigma })\left|\right|B_{1}\left|\right|. \end{array} \end{array}$$ (34)

As 0<ς<1, we get $1-\varsigma^{m-n}<1$. Therefore,

$$\begin{array}{c} \begin{array}{c} \left|\right|B_{m}-B_{n}\left|\right|\leq \frac{\varsigma^{n}}{1-\varsigma }max_{\Pi \in J}\left|\right|B_{1}\left|\right|. \end{array} \end{array}$$ (35)

Since $\left|\right|B_{1}\left||<\infty ,\;\;|\right|B_{m}-B_{n}\left|\right|\to 0$ when *n* → ∞. As a result, $B_{m}$ is a Cauchy sequence in H, implying that the series $B_{m}$ is convergent.

## 6 Applications

### 6.1 Example 1

Assume the nonlinear fractional mKdV equation as

$$\begin{array}{c} D_{\Pi }^{\mu }B(ℵ,\Pi )+6B^{2}\left(ℵ,\Pi \right)B_{ℵ}\left(ℵ,\Pi \right)+B_{ℵℵℵ}\left(ℵ,\Pi \right)=0,\;\;\;\;\;0<\mu \leq 1, \end{array}$$ (36)

with

$$\begin{array}{c} B\left(ℵ,0\right)=-\frac{2\kappa \;\text{exp}\left(\kappa ℵ\right)}{\text{exp}\left(2\kappa ℵ\right)+1}. \end{array}$$

Operating Definition 2.5 at n = 1, we may have

$$\begin{array}{c} \;E(\frac{\partial^{\mu }B}{\partial \Pi^{\mu }})=E[-6B^{2}\left(ℵ,\Pi \right)B_{ℵ}\left(ℵ,\Pi \right)-B_{ℵℵℵ}\left(ℵ,\Pi \right)]. \end{array}$$ (37)

Thus, we obtain

$$\begin{array}{c} \;\frac{1}{u^{\mu }}\left\{M\left(u\right)-u^{2}B\left(ℵ,0\right)\right\}=E[-6B^{2}\left(ℵ,\Pi \right)B_{ℵ}\left(ℵ,\Pi \right)-B_{ℵℵℵ}\left(ℵ,\Pi \right)], \end{array}$$ (38)

$$\begin{array}{c} \;M\left(u\right)=uB\left(ℵ,0\right)+u^{\mu }E[-6B^{2}\left(ℵ,\Pi \right)B_{ℵ}\left(ℵ,\Pi \right)-B_{ℵℵℵ}\left(ℵ,\Pi \right)]. \end{array}$$ (39)

On operating inverse ET, we may have

$$\begin{array}{c} \;\begin{array}{cc} & B\left(ℵ,\Pi \right)=B\left(ℵ,0\right)+E^{-1}[u^{\mu }\{E[-6B^{2}\left(ℵ,\Pi \right)B_{ℵ}\left(ℵ,\Pi \right)-B_{ℵℵℵ}\left(ℵ,\Pi \right)]\}],\\ & B\left(ℵ,\Pi \right)=-\frac{2\kappa \;\text{exp}\left(\kappa ℵ\right)}{\text{exp}\left(2\kappa ℵ\right)+1}+E^{-1}[u^{\mu }\{E[-6B^{2}\left(ℵ,\Pi \right)B_{ℵ}\left(ℵ,\Pi \right)-B_{ℵℵℵ}\left(ℵ,\Pi \right)]\}]. \end{array} \end{array}$$ (40)

By utilizing HPM, we may obtain

$$\begin{array}{c} \;\begin{array}{cc} & \sum_{k=0}^{\infty }\epsilon^{k}B_{k}\left(ℵ,\Pi \right)=(-\frac{2\kappa \;\text{exp}\left(\kappa ℵ\right)}{\text{exp}\left(2\kappa ℵ\right)+1})+\epsilon (E^{-1}[u^{\mu }E[-(\sum_{k=0}^{\infty }\epsilon^{k}H_{k}\left(B\right))-\\ & {\left(\sum_{k=0}^{\infty }\epsilon^{k}B_{k}\left(ℵ,\Pi \right)\right)}_{ℵℵℵ}]]). \end{array} \end{array}$$ (41)

The nonlinear term is illustrated as $H_{k}\left(B\right)$

$$\begin{array}{c} \sum_{k=0}^{\infty }\epsilon^{k}H_{k}\left(B\right)=B^{2}\left(ℵ,\Pi \right)B_{ℵ}\left(ℵ,\Pi \right). \end{array}$$ (42)

The nonlinear first components are given as

$$\begin{array}{c} \begin{array}{cc} & H_{0}\left(B\right)=B_{0}^{2}{\left(B_{0}\right)}_{ℵ},\\ & H_{1}\left(B\right)=B_{0}^{2}{\left(B_{1}\right)}_{ℵ}+2B_{0}B_{1}{\left(B_{0}\right)}_{ℵ},\\ & H_{2}\left(B\right)=B_{0}^{2}{\left(B_{2}\right)}_{ℵ}+2B_{0}B_{1}{\left(B_{1}\right)}_{ℵ}+\left(B_{1}^{2}+2B_{0}B_{2}\right){\left(B_{0}\right)}_{ℵ}, \end{array} \end{array}$$

Equating the *ϵ* coefficient on both sides as demonstrated below

$$\begin{array}{c} \;\begin{array}{cc} & \epsilon^{0}:B_{0}\left(ℵ,\Pi \right)=-\frac{2\kappa \;\text{exp}\left(\kappa ℵ\right)}{\text{exp}\left(2\kappa ℵ\right)+1},\\ & \epsilon^{1}:B_{1}\left(ℵ,\Pi \right)=E^{-1}(u^{\mu }E[-{\left(B_{0}\right)}_{ℵℵℵ}-H_{0}\left(B\right)])=-\frac{2\kappa^{4}\;\text{exp}\left(\kappa ℵ\right)\left(\text{exp}\left(2\kappa ℵ\right)-1\right)}{{\left(\text{exp}\left(2\kappa ℵ\right)+1\right)}^{2}}\frac{\Pi^{\mu }}{\Gamma (\mu +1)},\\ & \epsilon^{2}:B_{2}\left(ℵ,\Pi \right)=E^{-1}(u^{\mu }E[-{\left(B_{1}\right)}_{ℵℵℵ}-H_{1}\left(B\right)])=\\ & -\frac{\kappa^{7}\;\text{exp}\left(\kappa ℵ\right)\left(\text{exp}\left(4\kappa ℵ\right)-6\;\text{exp}\left(2\kappa ℵ\right)-1\right)}{{\left(\text{exp}\left(2\kappa ℵ\right)+1\right)}^{3}}\frac{\Pi^{2\mu }}{\Gamma (2\mu +1)},\\ & \epsilon^{3}:B_{3}\left(ℵ,\Pi \right)=E^{-1}(u^{\mu }E[-{\left(B_{2}\right)}_{ℵℵℵ}-H_{2}\left(B\right)])=-\frac{2\kappa^{10}\;\text{exp}\left(\kappa ℵ\right)}{{\left(\text{exp}\left(2\kappa ℵ\right)+1\right)}^{6}}\\ & \frac{(-165\;\text{exp}\left(8\kappa ℵ\right)+\text{exp}\left(10\kappa ℵ\right)+794\;\text{exp}\left(6\kappa ℵ\right)-794\;\text{exp}\left(4\kappa ℵ\right)+165\;\text{exp}\left(2\kappa ℵ\right)-1)\Pi^{3\mu }}{\Gamma (3\mu +1)}\\ & +\frac{48\kappa^{10}\;\text{exp}\left(\kappa ℵ\right)(17\;\text{exp}\left(6\kappa ℵ\right)-3\;\text{exp}\left(8\kappa ℵ\right)-17\;\text{exp}\left(4\kappa ℵ\right)+3\;\text{exp}\left(2\kappa ℵ\right))\Gamma \left(2\mu +1\right)}{{\left(\text{exp}\left(2\kappa ℵ\right)+1\right)}^{6}\Gamma {\left(\mu +1\right)}^{2}}\\ & \frac{\Pi^{3\mu }}{\Gamma (3\mu +1)}.\\ & \vdots \end{array} \end{array}$$

Hence, our approximate solution may be written in series form as

$$\begin{array}{c} \;\begin{array}{cc} & B\left(ℵ,\Pi \right)=B_{0}\left(ℵ,\Pi \right)+B_{1}\left(ℵ,\Pi \right)+B_{2}\left(ℵ,\Pi \right)+B_{3}\left(ℵ,\Pi \right)+\cdots \\ & B\left(ℵ,\Pi \right)=-\frac{2\kappa \;\text{exp}\left(\kappa ℵ\right)}{\text{exp}\left(2\kappa ℵ\right)+1}-\frac{2\kappa^{4}\;\text{exp}\left(\kappa ℵ\right)\left(\text{exp}\left(2\kappa ℵ\right)-1\right)}{{\left(\text{exp}\left(2\kappa ℵ\right)+1\right)}^{2}}\frac{\Pi^{\mu }}{\Gamma (\mu +1)}-\\ & \frac{\kappa^{7}\;\text{exp}\left(\kappa ℵ\right)\left(\text{exp}\left(4\kappa ℵ\right)-6\;\text{exp}\left(2\kappa ℵ\right)-1\right)}{{\left(\text{exp}\left(2\kappa ℵ\right)+1\right)}^{3}}\frac{\Pi^{2\mu }}{\Gamma (2\mu +1)}-\frac{2\kappa^{10}\;\text{exp}\left(\kappa ℵ\right)}{{\left(\text{exp}\left(2\kappa ℵ\right)+1\right)}^{6}}\\ & \frac{(-165\;\text{exp}\left(8\kappa ℵ\right)+\text{exp}\left(10\kappa ℵ\right)+794\;\text{exp}\left(6\kappa ℵ\right)-794\;\text{exp}\left(4\kappa ℵ\right)+165\;\text{exp}\left(2\kappa ℵ\right)-1)\Pi^{3\mu }}{\Gamma (3\mu +1)}\\ & +\frac{48\kappa^{10}\;\text{exp}\left(\kappa ℵ\right)(17\;\text{exp}\left(6\kappa ℵ\right)-3\;\text{exp}\left(8\kappa ℵ\right)-17\;\text{exp}\left(4\kappa ℵ\right)+3\;\text{exp}\left(2\kappa ℵ\right))\Gamma \left(2\mu +1\right)}{{\left(\text{exp}\left(2\kappa ℵ\right)+1\right)}^{6}\Gamma {\left(\mu +1\right)}^{2}}\\ & \frac{\Pi^{3\mu }}{\Gamma (3\mu +1)}+\cdots \end{array} \end{array}$$

#### Implementation of the ETDM

Operating Definition 2.5 at n = 1, we may have

$$\begin{array}{c} E\{\frac{\partial^{\mu }B}{\partial \Pi^{\mu }}\}=E[-6B^{2}\left(ℵ,\Pi \right)B_{ℵ}\left(ℵ,\Pi \right)-B_{ℵℵℵ}\left(ℵ,\Pi \right)]. \end{array}$$ (43)

Thus, we obtain

$$\begin{array}{c} \frac{1}{u^{\mu }}\left\{M\left(u\right)-u^{2}B\left(ℵ,0\right)\right\}=E[-6B^{2}\left(ℵ,\Pi \right)B_{ℵ}\left(ℵ,\Pi \right)-B_{ℵℵℵ}\left(ℵ,\Pi \right)], \end{array}$$ (44)

$$\begin{array}{c} M\left(u\right)=u^{2}B\left(ℵ,0\right)+u^{\mu }E[-6B^{2}\left(ℵ,\Pi \right)B_{ℵ}\left(ℵ,\Pi \right)-B_{ℵℵℵ}\left(ℵ,\Pi \right)]. \end{array}$$ (45)

On operating inverse ET, we may have

$$\begin{array}{c} \;\begin{array}{cc} & B\left(ℵ,\Pi \right)=B\left(ℵ,0\right)+E^{-1}[u^{\mu }\{E[-6B^{2}\left(ℵ,\Pi \right)B_{ℵ}\left(ℵ,\Pi \right)-B_{ℵℵℵ}\left(ℵ,\Pi \right)]\}],\\ & B\left(ℵ,\Pi \right)=-\frac{2\kappa \;\text{exp}\left(\kappa ℵ\right)}{\text{exp}\left(2\kappa ℵ\right)+1}+E^{-1}[u^{\mu }\{E[-6B^{2}\left(ℵ,\Pi \right)B_{ℵ}\left(ℵ,\Pi \right)-B_{ℵℵℵ}\left(ℵ,\Pi \right)]\}]. \end{array} \end{array}$$ (46)

Thus, the solution may be written in an infinite series form as

$$\begin{array}{c} B\left(ℵ,\Pi \right)=\sum_{m=0}^{\infty }B_{m}\left(ℵ,\Pi \right). \end{array}$$ (47)

The nonlinear terms are computed as $B^{2}\left(ℵ,\Pi \right)B_{ℵ}\left(ℵ,\Pi \right)=\sum_{m=0}^{\infty }A_{m}$

$$\begin{array}{c} \begin{array}{cc} & \sum_{m=0}^{\infty }B_{m}\left(ℵ,\Pi \right)=(-\frac{2\kappa \;\text{exp}\left(\kappa ℵ\right)}{\text{exp}\left(2\kappa ℵ\right)+1})-E^{-1}[u^{\mu }\{E[6\sum_{m=0}^{\infty }A_{m}+B_{ℵℵℵ}\left(ℵ,\Pi \right)]\}], \end{array} \end{array}$$ (48)

The nonlinear first components are given as

$$\begin{array}{c} \begin{array}{cc} & A_{0}=B_{0}^{2}{\left(B_{0}\right)}_{ℵ},\\ & A_{1}=B_{0}^{2}{\left(B_{1}\right)}_{ℵ}+2B_{0}B_{1}{\left(B_{0}\right)}_{ℵ},\\ & A_{2}=B_{0}^{2}{\left(B_{2}\right)}_{ℵ}+2B_{0}B_{1}{\left(B_{1}\right)}_{ℵ}+\left(B_{1}^{2}+2B_{0}B_{2}\right){\left(B_{0}\right)}_{ℵ}, \end{array} \end{array}$$

On equating both sides

$$\begin{array}{c} B_{0}\left(ℵ,\Pi \right)=-\frac{2\kappa \;\text{exp}\left(\kappa ℵ\right)}{\text{exp}\left(2\kappa ℵ\right)+1}. \end{array}$$

At *m* = 0,

$$\begin{array}{c} B_{1}\left(ℵ,\Pi \right)=-\frac{2\kappa^{4}\;\text{exp}\left(\kappa ℵ\right)\left(\text{exp}\left(2\kappa ℵ\right)-1\right)}{{\left(\text{exp}\left(2\kappa ℵ\right)+1\right)}^{2}}\frac{\Pi^{\mu }}{\Gamma (\mu +1)}. \end{array}$$

At *m* = 1

$$\begin{array}{c} \begin{array}{cc} & B_{2}\left(ℵ,\Pi \right)=-\frac{\kappa^{7}\;\text{exp}\left(\kappa ℵ\right)\left(\text{exp}\left(4\kappa ℵ\right)-6\;\text{exp}\left(2\kappa ℵ\right)-1\right)}{{\left(\text{exp}\left(2\kappa ℵ\right)+1\right)}^{3}}\frac{\Pi^{2\mu }}{\Gamma (2\mu +1)}. \end{array} \end{array}$$

At *m* = 2

$$\begin{array}{c} \begin{array}{cc} & B_{3}\left(ℵ,\Pi \right)=-\frac{2\kappa^{10}\;\text{exp}\left(\kappa ℵ\right)}{{\left(\text{exp}\left(2\kappa ℵ\right)+1\right)}^{6}}\\ & \frac{(-165\;\text{exp}\left(8\kappa ℵ\right)+\text{exp}\left(10\kappa ℵ\right)+794\;\text{exp}\left(6\kappa ℵ\right)-794\;\text{exp}\left(4\kappa ℵ\right)+165\;\text{exp}\left(2\kappa ℵ\right)-1)\Pi^{3\mu }}{\Gamma (3\mu +1)}\\ & +\frac{48\kappa^{10}\;\text{exp}\left(\kappa ℵ\right)(17\;\text{exp}\left(6\kappa ℵ\right)-3\;\text{exp}\left(8\kappa ℵ\right)-17\;\text{exp}\left(4\kappa ℵ\right)+3\;\text{exp}\left(2\kappa ℵ\right))\Gamma \left(2\mu +1\right)}{{\left(\text{exp}\left(2\kappa ℵ\right)+1\right)}^{6}\Gamma {\left(\mu +1\right)}^{2}}\\ & \frac{\Pi^{3\mu }}{\Gamma (3\mu +1)}. \end{array} \end{array}$$

Hence, our approximate solution may be written in series form as

$$\begin{array}{c} B\left(ℵ,\Pi \right)=\sum_{m=0}^{\infty }B_{m}\left(ℵ,\Pi \right)=B_{0}\left(ℵ,\Pi \right)+B_{1}\left(ℵ,\Pi \right)+B_{2}\left(ℵ,\Pi \right)+B_{3}\left(ℵ,\Pi \right)+\cdots \end{array}$$

$$\begin{array}{c} \begin{array}{cc} & B\left(ℵ,\Pi \right)=-\frac{2\kappa \;\text{exp}\left(\kappa ℵ\right)}{\text{exp}\left(2\kappa ℵ\right)+1}-\frac{2\kappa^{4}\;\text{exp}\left(\kappa ℵ\right)\left(\text{exp}\left(2\kappa ℵ\right)-1\right)}{{\left(\text{exp}\left(2\kappa ℵ\right)+1\right)}^{2}}\frac{\Pi^{\mu }}{\Gamma (\mu +1)}-\\ & \frac{\kappa^{7}\;\text{exp}\left(\kappa ℵ\right)\left(\text{exp}\left(4\kappa ℵ\right)-6\;\text{exp}\left(2\kappa ℵ\right)-1\right)}{{\left(\text{exp}\left(2\kappa ℵ\right)+1\right)}^{3}}\frac{\Pi^{2\mu }}{\Gamma (2\mu +1)}-\frac{2\kappa^{10}\;\text{exp}\left(\kappa ℵ\right)}{{\left(\text{exp}\left(2\kappa ℵ\right)+1\right)}^{6}}\\ & \frac{(-165\;\text{exp}\left(8\kappa ℵ\right)+\text{exp}\left(10\kappa ℵ\right)+794\;\text{exp}\left(6\kappa ℵ\right)-794\;\text{exp}\left(4\kappa ℵ\right)+165\;\text{exp}\left(2\kappa ℵ\right)-1)\Pi^{3\mu }}{\Gamma (3\mu +1)}\\ & +\frac{48\kappa^{10}\;\text{exp}\left(\kappa ℵ\right)(17\;\text{exp}\left(6\kappa ℵ\right)-3\;\text{exp}\left(8\kappa ℵ\right)-17\;\text{exp}\left(4\kappa ℵ\right)+3\;\text{exp}\left(2\kappa ℵ\right))\Gamma \left(2\mu +1\right)}{{\left(\text{exp}\left(2\kappa ℵ\right)+1\right)}^{6}\Gamma {\left(\mu +1\right)}^{2}}\\ & \frac{\Pi^{3\mu }}{\Gamma (3\mu +1)}+\cdots \end{array} \end{array}$$

By choosing *μ* = 1 we may have the following:

$$\begin{array}{c} \;B\left(ℵ,\Pi \right)=-\frac{2\kappa \;\text{exp}\left(\kappa \left(ℵ-\kappa^{2}\Pi \right)\right)}{\text{exp}\left(2\kappa \left(ℵ-\kappa^{2}\Pi \right)\right)+1}. \end{array}$$ (49)

### 6.2 Example 2

Assume the nonlinear fractional mKdV equation as

$$\begin{array}{c} D_{\Pi }^{\mu }B(ℵ,\Pi )+6B^{2}\left(ℵ,\Pi \right)B_{ℵ}\left(ℵ,\Pi \right)+B_{ℵℵℵ}\left(ℵ,\Pi \right)=0,\;\;\;\;\;0<\mu \leq 1, \end{array}$$ (50)

with

$$\begin{array}{c} B\left(ℵ,0\right)=\frac{4\;\text{exp}\left(ℵ\right)}{\text{exp}\left(2ℵ\right)+1}. \end{array}$$

Operating Definition 2.5 at n = 1, we may have

$$\begin{array}{c} \;E(\frac{\partial^{\mu }B}{\partial \Pi^{\mu }})=E[-6B^{2}\left(ℵ,\Pi \right)B_{ℵ}\left(ℵ,\Pi \right)-B_{ℵℵℵ}\left(ℵ,\Pi \right)]. \end{array}$$ (51)

Thus, we obtain

$$\begin{array}{c} \;\frac{1}{u^{\mu }}\left\{M\left(u\right)-u^{2}B\left(ℵ,0\right)\right\}=E[-6B^{2}\left(ℵ,\Pi \right)B_{ℵ}\left(ℵ,\Pi \right)-B_{ℵℵℵ}\left(ℵ,\Pi \right)], \end{array}$$ (52)

$$\begin{array}{c} \;M\left(u\right)=uB\left(ℵ,0\right)+u^{\mu }E[-6B^{2}\left(ℵ,\Pi \right)B_{ℵ}\left(ℵ,\Pi \right)-B_{ℵℵℵ}\left(ℵ,\Pi \right)]. \end{array}$$ (53)

On operating inverse ET, we may have

$$\begin{array}{c} \;\begin{array}{cc} & B\left(ℵ,\Pi \right)=B\left(ℵ,0\right)+E^{-1}[u^{\mu }\{E[-6B^{2}\left(ℵ,\Pi \right)B_{ℵ}\left(ℵ,\Pi \right)-B_{ℵℵℵ}\left(ℵ,\Pi \right)]\}],\\ & B\left(ℵ,\Pi \right)=\frac{4\;\text{exp}\left(ℵ\right)}{\text{exp}\left(2ℵ\right)+1}+E^{-1}[u^{\mu }\{E[-6B^{2}\left(ℵ,\Pi \right)B_{ℵ}\left(ℵ,\Pi \right)-B_{ℵℵℵ}\left(ℵ,\Pi \right)]\}]. \end{array} \end{array}$$ (54)

By utilizing HPM, we may obtain

$$\begin{array}{c} \;\begin{array}{cc} & \sum_{k=0}^{\infty }\epsilon^{k}B_{k}\left(ℵ,\Pi \right)=(\frac{4\;\text{exp}\left(ℵ\right)}{\text{exp}\left(2ℵ\right)+1})+\epsilon (E^{-1}[u^{\mu }E[-(\sum_{k=0}^{\infty }\epsilon^{k}H_{k}\left(B\right))-\\ & {\left(\sum_{k=0}^{\infty }\epsilon^{k}B_{k}\left(ℵ,\Pi \right)\right)}_{ℵℵℵ}]]). \end{array} \end{array}$$ (55)

The nonlinear term is illustrated as $H_{k}\left(B\right)$ as

$$\begin{array}{c} \sum_{k=0}^{\infty }\epsilon^{k}H_{k}\left(B\right)=B^{2}\left(ℵ,\Pi \right)B_{ℵ}\left(ℵ,\Pi \right). \end{array}$$ (56)

The nonlinear first components are given as

$$\begin{array}{c} \begin{array}{cc} & H_{0}\left(B\right)=B_{0}^{2}{\left(B_{0}\right)}_{ℵ},\\ & H_{1}\left(B\right)=B_{0}^{2}{\left(B_{1}\right)}_{ℵ}+2B_{0}B_{1}{\left(B_{0}\right)}_{ℵ},\\ & H_{2}\left(B\right)=B_{0}^{2}{\left(B_{2}\right)}_{ℵ}+2B_{0}B_{1}{\left(B_{1}\right)}_{ℵ}+\left(B_{1}^{2}+2B_{0}B_{2}\right){\left(B_{0}\right)}_{ℵ}, \end{array} \end{array}$$

Equating the *ϵ* coefficient on both sides as demonstrated below

$$\begin{array}{c} \;\begin{array}{cc} & \epsilon^{0}:B_{0}\left(ℵ,\Pi \right)=\frac{4\;\text{exp}\left(ℵ\right)}{\text{exp}\left(2ℵ\right)+1},\\ & \epsilon^{1}:B_{1}\left(ℵ,\Pi \right)=E^{-1}(u^{\mu }E[-{\left(B_{0}\right)}_{ℵℵℵ}-H_{0}\left(B\right)])=\frac{4\;\text{exp}\left(ℵ\right)}{{\left(\text{exp}\left(2ℵ\right)+1\right)}^{4}}(\text{exp}\left(6ℵ\right)+73\\ & \text{exp}\left(4ℵ\right)-73\;\text{exp}\left(2ℵ\right)-1)\frac{\Pi^{\mu }}{\Gamma (\mu +1)},\\ & \epsilon^{2}:B_{2}\left(ℵ,\Pi \right)=E^{-1}(u^{\mu }E[-{\left(B_{1}\right)}_{ℵℵℵ}-H_{1}\left(B\right)])=\frac{4\;\text{exp}\left(ℵ\right)}{{\left(\text{exp}\left(2ℵ\right)+1\right)}^{7}}(\text{exp}\left(12ℵ\right)+2158\\ & \text{exp}\left(10ℵ\right)+2863\;\text{exp}\left(8ℵ\right)-26236\;\text{exp}\left(6ℵ\right)+2863\;\text{exp}\left(4ℵ\right)+2158\;\text{exp}\left(2ℵ\right)+1)\frac{\Pi^{2\mu }}{\Gamma (2\mu +1)},\\ & \epsilon^{3}:B_{3}\left(ℵ,\Pi \right)=E^{-1}(u^{\mu }E[-{\left(B_{2}\right)}_{ℵℵℵ}-H_{2}\left(B\right)])=\frac{4\;\text{exp}\left(ℵ\right)}{{\left(\text{exp}\left(2ℵ\right)+1\right)}^{10}}(-1-8600804\\ & \text{exp}\left(12ℵ\right)-23219726\;\text{exp}\left(8ℵ\right)+23219726\;\text{exp}\left(10ℵ\right)-88340\;\text{exp}\left(4ℵ\right)+8600804\;\text{exp}\left(6ℵ\right)\\ & -58375\;\text{exp}\left(2ℵ\right)+\text{exp}\left(18ℵ\right)+88340\;\text{exp}\left(14ℵ\right)+58375\;\text{exp}\left(16ℵ\right))\frac{\Pi^{3\mu }}{\Gamma (3\mu +1)}+\\ & \frac{384\;\text{exp}\left(ℵ\right)\Gamma (2\mu +1)}{{\left(\text{exp}\left(2ℵ\right)+1\right)}^{10}\Gamma {\left(\mu +1\right)}^{2}}(715\;\text{exp}\left(14ℵ\right)+3\;\text{exp}\left(16ℵ\right)-152953\;\text{exp}\left(10ℵ\right)+34383\\ & \text{exp}\left(12ℵ\right)+152953\;\text{exp}\left(8ℵ\right)-34383\;\text{exp}\left(6ℵ\right)-715\;\text{exp}\left(4ℵ\right)-3\;\text{exp}\left(2ℵ\right))\frac{\Pi^{3\mu }}{\Gamma (3\mu +1)},\\ & \vdots \end{array} \end{array}$$

Hence, our approximate solution may be written in series form as

$$\begin{array}{c} \;\begin{array}{cc} & B\left(ℵ,\Pi \right)=B_{0}\left(ℵ,\Pi \right)+B_{1}\left(ℵ,\Pi \right)+B_{2}\left(ℵ,\Pi \right)+B_{3}\left(ℵ,\Pi \right)+\cdots \\ & B\left(ℵ,\Pi \right)=\frac{4\;\text{exp}\left(ℵ\right)}{\text{exp}\left(2ℵ\right)+1}+\frac{4\;\text{exp}\left(ℵ\right)}{{\left(\text{exp}\left(2ℵ\right)+1\right)}^{4}}\left(\text{exp}\left(6ℵ\right)+73\;\text{exp}\left(4ℵ\right)-73\;\text{exp}\left(2ℵ\right)-1\right)\\ & \frac{\Pi^{\mu }}{\Gamma (\mu +1)}+\frac{4\;\text{exp}\left(ℵ\right)}{{\left(\text{exp}\left(2ℵ\right)+1\right)}^{7}}(\text{exp}\left(12ℵ\right)+2158\;\text{exp}\left(10ℵ\right)+2863\;\text{exp}\left(8ℵ\right)-26236\\ & \text{exp}\left(6ℵ\right)+2863\;\text{exp}\left(4ℵ\right)+2158\;\text{exp}\left(2ℵ\right)+1)\frac{\Pi^{2\mu }}{\Gamma (2\mu +1)}+\frac{4\;\text{exp}\left(ℵ\right)}{{\left(\text{exp}\left(2ℵ\right)+1\right)}^{10}}(-1-8600804\\ & \text{exp}\left(12ℵ\right)-23219726\;\text{exp}\left(8ℵ\right)+23219726\;\text{exp}\left(10ℵ\right)-88340\;\text{exp}\left(4ℵ\right)+8600804\;\text{exp}\left(6ℵ\right)\\ & -58375\;\text{exp}\left(2ℵ\right)+\text{exp}\left(18ℵ\right)+88340\;\text{exp}\left(14ℵ\right)+58375\;\text{exp}\left(16ℵ\right))\frac{\Pi^{3\mu }}{\Gamma (3\mu +1)}+\\ & \frac{384\;\text{exp}\left(ℵ\right)\Gamma (2\mu +1)}{{\left(\text{exp}\left(2ℵ\right)+1\right)}^{10}\Gamma {\left(\mu +1\right)}^{2}}(715\;\text{exp}\left(14ℵ\right)+3\;\text{exp}\left(16ℵ\right)-152953\;\text{exp}\left(10ℵ\right)+34383\\ & \text{exp}\left(12ℵ\right)+152953\;\text{exp}\left(8ℵ\right)-34383\;\text{exp}\left(6ℵ\right)-715\;\text{exp}\left(4ℵ\right)-3\;\text{exp}\left(2ℵ\right))\frac{\Pi^{3\mu }}{\Gamma (3\mu +1)}+\cdots \end{array} \end{array}$$

#### Implementation of the ETDM

Operating Definition 2.5 at n = 1, we may have

$$\begin{array}{c} E\{\frac{\partial^{\mu }B}{\partial \Pi^{\mu }}\}=E[-6B^{2}\left(ℵ,\Pi \right)B_{ℵ}\left(ℵ,\Pi \right)-B_{ℵℵℵ}\left(ℵ,\Pi \right)]. \end{array}$$ (57)

Thus, we obtain

$$\begin{array}{c} \frac{1}{u^{\mu }}\left\{M\left(u\right)-u^{2}B\left(ℵ,0\right)\right\}=E[-6B^{2}\left(ℵ,\Pi \right)B_{ℵ}\left(ℵ,\Pi \right)-B_{ℵℵℵ}\left(ℵ,\Pi \right)], \end{array}$$ (58)

$$\begin{array}{c} M\left(u\right)=u^{2}B\left(ℵ,0\right)+u^{\mu }E[-6B^{2}\left(ℵ,\Pi \right)B_{ℵ}\left(ℵ,\Pi \right)-B_{ℵℵℵ}\left(ℵ,\Pi \right)]. \end{array}$$ (59)

On operating inverse ET, we may have

$$\begin{array}{c} \;\begin{array}{cc} & B\left(ℵ,\Pi \right)=B\left(ℵ,0\right)+E^{-1}[u^{\mu }\{E[-6B^{2}\left(ℵ,\Pi \right)B_{ℵ}\left(ℵ,\Pi \right)-B_{ℵℵℵ}\left(ℵ,\Pi \right)]\}],\\ & B\left(ℵ,\Pi \right)=\frac{4\;\text{exp}\left(ℵ\right)}{\text{exp}\left(2ℵ\right)+1}+E^{-1}[u^{\mu }\{E[-6B^{2}\left(ℵ,\Pi \right)B_{ℵ}\left(ℵ,\Pi \right)-B_{ℵℵℵ}\left(ℵ,\Pi \right)]\}]. \end{array} \end{array}$$ (60)

Thus, the solution in series form is as

$$\begin{array}{c} B\left(ℵ,\Pi \right)=\sum_{m=0}^{\infty }B_{m}\left(ℵ,\Pi \right). \end{array}$$ (61)

Thus, the solution may be written in an infinite series form as $B^{2}\left(ℵ,\Pi \right)B_{ℵ}\left(ℵ,\Pi \right)=\sum_{m=0}^{\infty }A_{m}$

$$\begin{array}{c} \begin{array}{cc} & \sum_{m=0}^{\infty }B_{m}\left(ℵ,\Pi \right)=(\frac{4\;\text{exp}\left(ℵ\right)}{\text{exp}\left(2ℵ\right)+1})-E^{-1}[u^{\mu }\{E[6\sum_{m=0}^{\infty }A_{m}+B_{ℵℵℵ}\left(ℵ,\Pi \right)]\}]. \end{array} \end{array}$$ (62)

The nonlinear first components are given as

$$\begin{array}{c} \begin{array}{cc} & A_{0}=B_{0}^{2}{\left(B_{0}\right)}_{ℵ},\\ & A_{1}=B_{0}^{2}{\left(B_{1}\right)}_{ℵ}+2B_{0}B_{1}{\left(B_{0}\right)}_{ℵ},\\ & A_{2}=B_{0}^{2}{\left(B_{2}\right)}_{ℵ}+2B_{0}B_{1}{\left(B_{1}\right)}_{ℵ}+\left(B_{1}^{2}+2B_{0}B_{2}\right){\left(B_{0}\right)}_{ℵ}, \end{array} \end{array}$$

On equating both sides

$$\begin{array}{c} B_{0}\left(ℵ,\Pi \right)=\frac{4\;\text{exp}\left(ℵ\right)}{\text{exp}\left(2ℵ\right)+1}. \end{array}$$

At *m* = 0,

$$\begin{array}{c} B_{1}\left(ℵ,\Pi \right)=\frac{4\;\text{exp}\left(ℵ\right)}{{\left(\text{exp}\left(2ℵ\right)+1\right)}^{4}}\left(\text{exp}\left(6ℵ\right)+73\;\text{exp}\left(4ℵ\right)-73\;\text{exp}\left(2ℵ\right)-1\right)\frac{\Pi^{\mu }}{\Gamma (\mu +1)}. \end{array}$$

At *m* = 1

$$\begin{array}{c} \begin{array}{cc} & B_{2}\left(ℵ,\Pi \right)=\frac{2\;\text{exp}\left(ℵ\right)}{{\left(\text{exp}\left(2ℵ\right)+1\right)}^{7}}(\text{exp}\left(12ℵ\right)+2158\;\text{exp}\left(10ℵ\right)+2863\;\text{exp}\left(8ℵ\right)-26236\;\text{exp}\left(6ℵ\right)\\ & +2863\;\text{exp}\left(4ℵ\right)+2158\;\text{exp}\left(2ℵ\right)+1)\frac{\Pi^{2\mu }}{\Gamma (2\mu +1)}. \end{array} \end{array}$$

At *m* = 2

$$\begin{array}{c} \begin{array}{cc} & B_{3}\left(ℵ,\Pi \right)=\frac{4\;\text{exp}\left(ℵ\right)}{{\left(\text{exp}\left(2ℵ\right)+1\right)}^{10}}(-1-8600804\;\text{exp}\left(12ℵ\right)-23219726\;\text{exp}\left(8ℵ\right)+23219726\\ & \text{exp}\left(10ℵ\right)-88340\;\text{exp}\left(4ℵ\right)+8600804\;\text{exp}\left(6ℵ\right)-58375\;\text{exp}\left(2ℵ\right)+\text{exp}\left(18ℵ\right)+88340\\ & \text{exp}\left(14ℵ\right)+58375\;\text{exp}\left(16ℵ\right))\frac{\Pi^{3\mu }}{\Gamma (3\mu +1)}+\frac{384\;\text{exp}\left(ℵ\right)\Gamma (2\mu +1)}{{\left(\text{exp}\left(2ℵ\right)+1\right)}^{10}\Gamma {\left(\mu +1\right)}^{2}}(715\;\text{exp}\left(14ℵ\right)+\\ & 3\;\text{exp}\left(16ℵ\right)-152953\;\text{exp}\left(10ℵ\right)+34383\;\text{exp}\left(12ℵ\right)+152953\;\text{exp}\left(8ℵ\right)-34383\;\text{exp}\left(6ℵ\right)-\\ & 715\;\text{exp}\left(4ℵ\right)-3\;\text{exp}\left(2ℵ\right))\frac{\Pi^{3\mu }}{\Gamma (3\mu +1)}. \end{array} \end{array}$$

Hence, our approximate solution may be written in series form as

$$\begin{array}{c} B\left(ℵ,\Pi \right)=\sum_{m=0}^{\infty }B_{m}\left(ℵ,\Pi \right)=B_{0}\left(ℵ,\Pi \right)+B_{1}\left(ℵ,\Pi \right)+B_{2}\left(ℵ,\Pi \right)+B_{3}\left(ℵ,\Pi \right)+\cdots \end{array}$$

$$\begin{array}{c} \begin{array}{cc} & B\left(ℵ,\Pi \right)=\frac{4\;\text{exp}\left(ℵ\right)}{\text{exp}\left(2ℵ\right)+1}+\frac{4\;\text{exp}\left(ℵ\right)}{{\left(\text{exp}\left(2ℵ\right)+1\right)}^{4}}\left(\text{exp}\left(6ℵ\right)+73\;\text{exp}\left(4ℵ\right)-73\;\text{exp}\left(2ℵ\right)-1\right)\\ & \frac{\Pi^{\mu }}{\Gamma (\mu +1)}+\frac{4\;\text{exp}\left(ℵ\right)}{{\left(\text{exp}\left(2ℵ\right)+1\right)}^{7}}(\text{exp}\left(12ℵ\right)+2158\;\text{exp}\left(10ℵ\right)+2863\;\text{exp}\left(8ℵ\right)-26236\\ & \text{exp}\left(6ℵ\right)+2863\;\text{exp}\left(4ℵ\right)+2158\;\text{exp}\left(2ℵ\right)+1)\frac{\Pi^{2\mu }}{\Gamma (2\mu +1)}+\frac{4\;\text{exp}\left(ℵ\right)}{{\left(\text{exp}\left(2ℵ\right)+1\right)}^{10}}(-1-8600804\\ & \text{exp}\left(12ℵ\right)-23219726\;\text{exp}\left(8ℵ\right)+23219726\;\text{exp}\left(10ℵ\right)-88340\;\text{exp}\left(4ℵ\right)+8600804\;\text{exp}\left(6ℵ\right)\\ & -58375\;\text{exp}\left(2ℵ\right)+\text{exp}\left(18ℵ\right)+88340\;\text{exp}\left(14ℵ\right)+58375\;\text{exp}\left(16ℵ\right))\frac{\Pi^{3\mu }}{\Gamma (3\mu +1)}+\\ & \frac{384\;\text{exp}\left(ℵ\right)\Gamma (2\mu +1)}{{\left(\text{exp}\left(2ℵ\right)+1\right)}^{10}\Gamma {\left(\mu +1\right)}^{2}}(715\;\text{exp}\left(14ℵ\right)+3\;\text{exp}\left(16ℵ\right)-152953\;\text{exp}\left(10ℵ\right)+34383\\ & \text{exp}\left(12ℵ\right)+152953\;\text{exp}\left(8ℵ\right)-34383\;\text{exp}\left(6ℵ\right)-715\;\text{exp}\left(4ℵ\right)-3\;\text{exp}\left(2ℵ\right))\frac{\Pi^{3\mu }}{\Gamma (3\mu +1)}+\cdots \end{array} \end{array}$$

By choosing *μ* = 1 we may have

$$\begin{array}{c} \;B\left(ℵ,\Pi \right)=\frac{4(\text{exp}\left(\Pi -ℵ\right)+3\;\text{exp}\left(27\Pi -3ℵ\right)+3\;\text{exp}\left(29\Pi -5ℵ\right)+\text{exp}\left(55\Pi -7ℵ\right))}{1+4\;\text{exp}\left(2\Pi -2ℵ\right)+6\;\text{exp}\left(28\Pi -4ℵ\right)+4\;\text{exp}\left(54\Pi -6ℵ\right)+\text{exp}\left(56\Pi -8ℵ\right)}. \end{array}$$ (63)

## 7 Results and discussion

**Fig 1:**
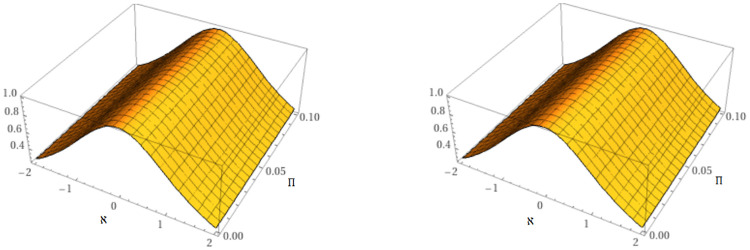
Behavior of the accurate and proposed methods solution of example 1.

In this study, two unique methods have been employed for examining the approximate solution of time-fractional mKdV equations. The suggested equations have exact analytical solutions that can be found using *Maple* software at any order for different space and time values by employing the Caputo derivative. The precise and analytical behavior of the suggested approaches is depicted in Fig 1. Fig 1 makes it evident that the subfigures are almost exact and in good agreement with one another for the particular situation *μ* = 1.

**Fig 2:**
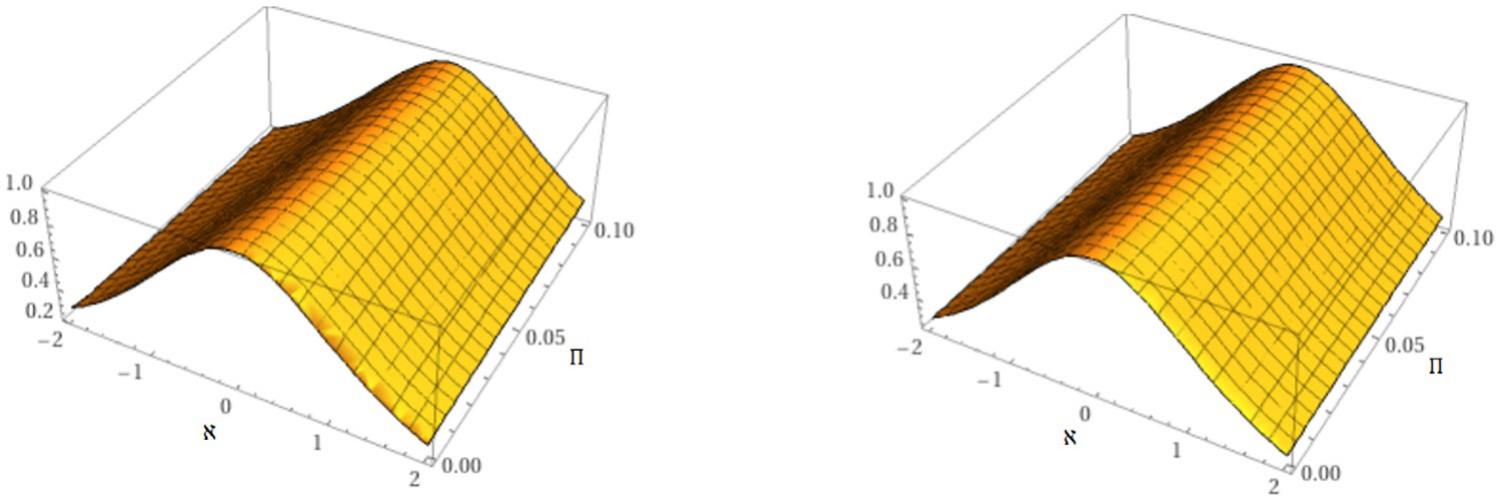
Behavior of the suggested methods solution at *μ* = 0.6 and *μ* = 0.8.

Fig 2 illustrates the mKdV equation’s dynamics at *μ* = 0.60 and *μ* = 0.8.

**Fig 3:**
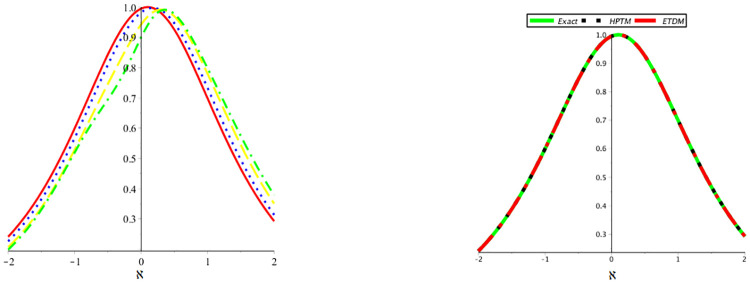
Behavior of the suggested methods solution at various orders of *μ* and comparison between accurate and suggested methods solution.

Fig 3 illustrates the mKdV equation’s 2D dynamics at numerous fractional orders *μ* = 0.40, 0.60, 0.80, 1 and comparison between accurate and suggested methods solution to problem 6.1.

Table 1 presents a comparison of the time-fractional mKdV equation solution with varying fractional order *μ*.

**Table 1:** Comparative analysis of the exact and proposed methods solution at different fractional orders.

ℵ	*μ* = 0.97	*μ* = 0.98	*μ* = 0.99	*μ* = 1(*appro*)	*μ* = 1(*exact*)
0.0	0.9999303847	0.9999376463	0.9999441593	0.9999500000	0.9999500020
0.1	0.9961055785	0.9960563210	0.9960090188	0.9959636219	0.9959636242
0.2	0.9825141342	0.9824107758	0.9823119795	0.9822175721	0.9822175738
0.3	0.9598118502	0.9596593019	0.9595137039	0.9593747680	0.9593747688
0.4	0.9290468421	0.9288519590	0.9286660977	0.9284888691	0.9284888698
0.5	0.8915470808	0.8913179545	0.8910995420	0.8908913695	0.8908913694
0.6	0.8487917566	0.8485369803	0.8482942007	0.8480628783	0.8480628778
0.7	0.8022874642	0.8020154743	0.8017563585	0.8015095309	0.8015095304
0.8	0.7534650985	0.7531836655	0.7529156073	0.7526603102	0.7526603096
0.9	0.7036059849	0.7033218807	0.7030513217	0.7027936822	0.7027936814
1.0	0.6537986827	0.6535175159	0.6532497893	0.6529948784	0.6529948774

Similarly, Table 2 presents a comparison of the time-fractional mKdV equation solution with varying fractional order *μ* in terms of absolute error.

**Table 2:** Comparative analysis of the proposed methods solution at different fractional orders in terms of absolute error.

ℵ	Our Methods error at *μ* = 0.97	Our Methods error at *μ* = 0.98	Our Methods error at *μ* = 0.99	Our Methods error at *μ* = 1
0.0	1.9617300000E-05	1.2355700000E-05	5.8427000000E-06	2.0000000000E-09
0.1	1.4195430000E-04	9.2696800000E-05	4.5394600000E-05	2.3000000000E-09
0.2	2.9656040000E-04	1.9320200000E-04	9.4405700000E-05	1.7000000000E-09
0.3	4.3708140000E-04	2.8453310000E-04	1.3893510000E-04	8.0000000000E-10
0.4	5.5797230000E-04	3.6308920000E-04	1.7722790000E-04	7.0000000000E-10
0.5	6.5571140000E-04	4.2658510000E-04	2.0817260000E-04	1.0000000000E-10
0.6	7.2887880000E-04	4.7410250000E-04	2.3132290000E-04	5.0000000000E-10
0.7	7.7793380000E-04	5.0594390000E-04	2.4682810000E-04	5.0000000000E-10
0.8	8.0478890000E-04	5.2335590000E-04	2.5529770000E-04	6.0000000000E-10
0.9	8.1230350000E-04	5.2819930000E-04	2.5764030000E-04	8.0000000000E-10
1.0	8.0380530000E-04	5.2263850000E-04	2.5491190000E-04	1.0000000000E-09

**Fig 4:**
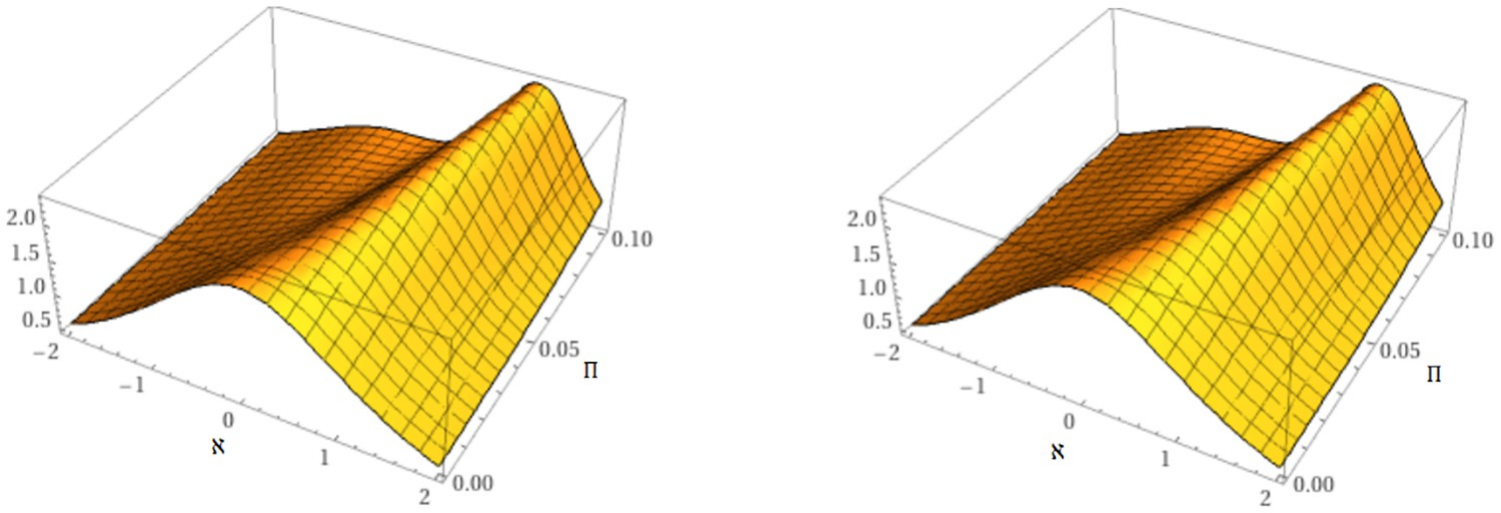
Behavior of the accurate and proposed methods solution of example 2.

The nature of the exact solution and the approximate solution of problem 6.2 at *μ* = 1 are examined in Fig 4.

**Fig 5:**
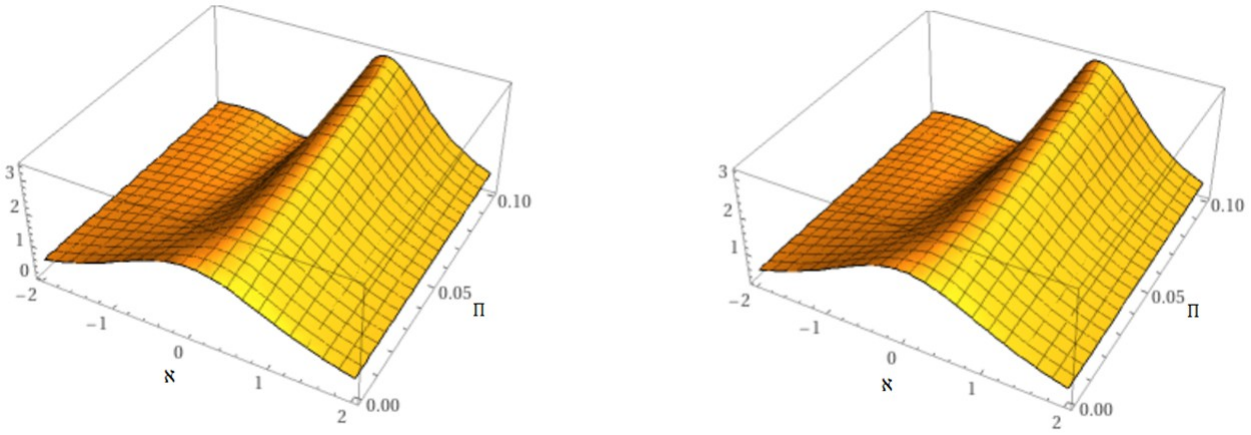
Behavior of the suggested methods solution at *μ* = 0.6 and *μ* = 0.8.

Fig 5 illustrates the mKdV equation’s dynamics at *μ* = 0.60 and *μ* = 0.8.

**Fig 6:**
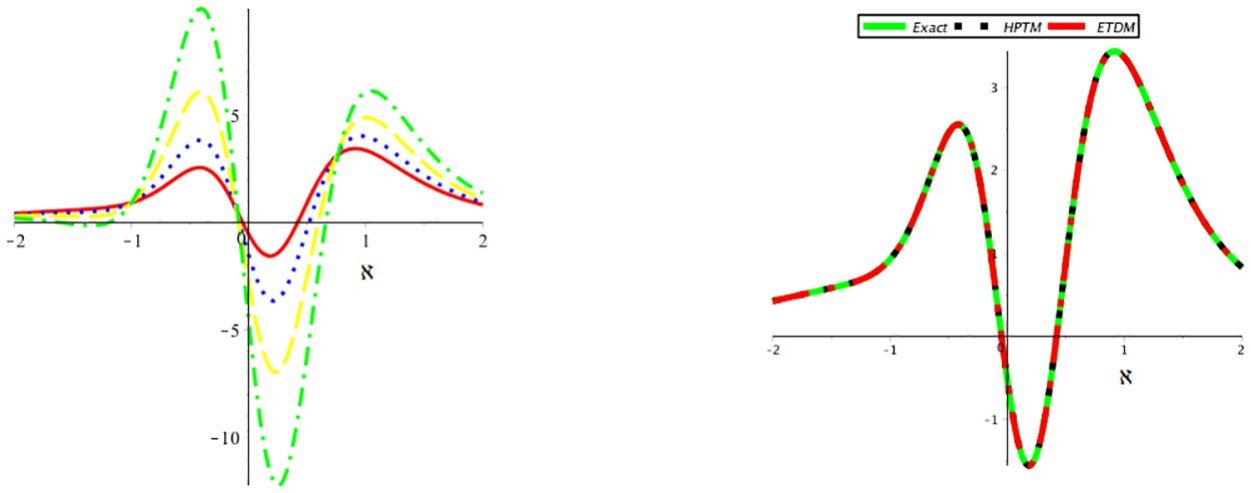
Behavior of the suggested methods solution at various orders of *μ* and comparison between accurate and suggested methods solution.

Furthermore, the approximate solution behavior at *μ* = 0.40, 0.60, 0.80, 1 and comparison between accurate and suggested methods solution is shown in Fig 6 to problem 6.2.

The approximate solutions closely resemble the exact solutions at *μ* = 1, as can be seen from the graphical solutions. Comparing the solutions to the time-fractional mKdV equation at various fractional orders *μ* is also displayed in Table 3.

**Table 3:** Comparative analysis of the exact and proposed methods solution at different fractional orders.

ℵ	*μ* = 0.97	*μ* = 0.98	*μ* = 0.99	*μ* = 1(*appro*)	*μ* = 1(*exact*)
0.0	1.9647746790	1.9684490100	1.9717446080	1.9747000000	1.9751019100
0.1	1.9982243380	1.9998840860	2.0012385380	2.0023269240	2.0026965600
0.2	2.0121894970	2.0115667920	2.0107558190	2.0097909320	2.0100225490
0.3	2.0034579320	2.0005536450	1.9976061310	1.9946400360	1.9946899760
0.4	1.9704117660	1.9655075890	1.9607101980	1.9560316870	1.9559268280
0.5	1.9135842790	1.9071794340	1.9010134010	1.8950853880	1.8948945100
0.6	1.8356654960	1.8283648410	1.8214008300	1.8147615890	1.8145559940
0.7	1.7409728400	1.7333693860	1.7261597030	1.7193239250	1.7191516490
0.8	1.6346268010	1.6272140460	1.6202137970	1.6136016130	1.6134819890
0.9	1.5217327400	1.5148616780	1.5083913840	1.5022958750	1.5022272470
1.0	1.4067809920	1.4006573080	1.3949021630	1.3894903000	1.3894609130

Using the applied methodologies, numerical experiments have been carried out for different values of *μ*, successfully yielding good results for the problems under study. Therefore, it is clear that adding new approximation terms can reduce overall errors. One can get a good approximation with the exact solution by employing a limited number of terms. The given numerical simulations guarantee the accuracy of the results. Tables provide significantly better results compared to the exact solution. In summary, we believe that the techniques we implemented are reliable and relevant to interdisciplinary study classifications, such as fractional-order nonlinear scientific methodologies, which develops our understanding of nonlinear compound phenomena in science and innovation domains.

## 8 Conclusion

This work has investigated in detail two distinct approaches for solving mKdV equations: the ETDM and the HPTM. The fractional-order solutions give different dynamics for different fractional orders of the derivative. The task can be achieved easily and efficiently with analytical solutions, avoiding the intricate computations required by numerical methods. Overall, the scholars can now select the non-integer order problem whose solution is similar or very close to the outcomes of any physical experiment. The related solutions observed under the Caputo operator validate the significant dynamics of the problems presented. The given graphical behavior and numerical simulation of the problems demonstrate the dependability of the applied analytical techniques. Additionally, a comparison between exact and approximate solutions is provided. Tables and graphs displaying the computed study results have been achieved. These methods have proven to be both efficient and practical, making them applicable to a wide range of nonlinear problems. In light of the benefits offered by the existing operator, it will be highly appreciated to expand in the future to include additional operators and methodologies. The presented solutions were found to be easy to implement, making them appropriate for handling any physical issues that may arise in the fields of engineering and science. Lastly, the methods provided novel insights into the study of non-linear phenomena in plasma physics, such as soliton, rogue, and shock waves. In the near future, we plan to employ these approaches, including the Atangana-Baleanu derivative, to solve other partial differential equations that make use of fractional calculus and fractal theory.
